# Editorial: Monocot phylogenetics and trait evolution

**DOI:** 10.3389/fpls.2022.1076169

**Published:** 2022-11-10

**Authors:** Margarita V. Remizowa, Sean W. Graham, Paula J. Rudall

**Affiliations:** ^1^ Department of Higher Plants, Faculty of Biology, M.V. Lomonosov Moscow State University, Moscow, Russia; ^2^ Department of Botany, University of British Columbia, Vancouver, BC, Canada; ^3^ Jodrell Laboratory, Royal Botanic Gardens Kew, London, United Kingdom

**Keywords:** monocotyledons, monocot systematics, trait evolution, phylogenomics, plant architecture

According to most recent estimates, the monocot lineage diverged around 140 Ma and diversified relatively rapidly into the species groupings that we now classify into about 77 families in 12 orders (Givnish et al., 2018). From the late 20th century onward, monocot classification was transformed by the use of cladistic methodology to evaluate large suites of characters, both morphological and molecular. A pioneering series of morphology-based studies by the Scandinavian botanist Rolf Dahlgren and his co-workers in the 1980s was rapidly augmented by the advent of molecular phylogenetics and an ongoing monocot conference series in the 1990s (Dahlgren et al., 1985; Rudall, 2017). Considerable progress was made in early analyses that used one or a few genes, typically plastid genes (*atpB*, *rbcL*, *matK*, etc), nuclear ribosomal regions (18S rDNA, ITS), or combinations of these. However, numerous branches in the monocot tree of life have remained poorly understood. More recently, advances in DNA sequencing technologies (next-generation or high-throughput sequencing), coupled with increasingly automated analytical techniques, have allowed us to address some of the more recalcitrant outstanding issues, improve our understanding of relationships at various taxonomic levels, and to build species trees, for example based on gene trees derived from genome-scale data sets (e.g., Baker et al., 2021). This enhanced phylogenetic context provides the basis for a fresh look at trait evolution, and helps to improve our understanding of associations between genes and functional traits in both a systematic and ecological context.

## Role of phylogenomic studies in monocot systematics

Molecular phylogenetic and phylogenomic studies have provided a generally strong consensus on the broader higher-level relationships of the orders and families of monocots, but support for some relationships remains weak or conflicted. Confident resolution of ambiguous branches in plant phylogeny matters for downstream analyses of traits, timelines and geography. The study by Li et al. uses whole-plastome data to infer relationships within tribe Lilieae (Liliaceae, order Liliales), which contains many bulbous species that specialise in seasonally dry habitats. In turn, their generally well-supported tree estimate corroborates an Eocene origin for the tribe in the high altitude Qinghai-Tibet plateau, followed by subsequent radiations in the Himalayas and Hengduan mountains.

A recent large-scale plastome-based phylogeny examining monocot family-level relationships laid the groundwork for understanding higher-level monocot phylogeny (Givnish et al., 2018). However, this study was based on evidence from one linkage group—effectively a single “coalescent” gene (Doyle, 2022). In the present Research Topic, Timilsina et al. evaluate species-level relationships in all 12 monocot orders and 72 of 77 families, comparing results from 602 conserved single-copy (CSC) genes and 1375 benchmarking single-copy ortholog (BUSCO) genes, all extracted from transcriptomic and genomic datasets. These two gene sets provide independent phylogenetic estimates based on genes distributed throughout the nuclear genome. The specific genes present in the BUSCO and CSC data sets had partial overlap (~20% of genes), but the two genes sets have indistinguishable functional biases based on functional annotation clustering. Although the majority of BUSCO genes are not strictly single-copy in monocots, they yield tree inferences that are highly congruent with those based on CSC genes in analyses that accommodate the multi-species coalescent (MSC) process across individual nuclear gene-tree histories.

The Timilsina et al. study both broadly corroborates previous studies based on plastid-based evidence, and resolves multiple previously contentious branches with strong support here. For example, concerning the earliest evolutionary splits in monocot phylogeny, Timilsina et al. recovered the family Tofieldiaceae as the sister group of all remaining families of Alismatales rather than Araceae (alternative arrangements of these clades had been found with weak support in previous plastid-based analyses, e.g., Ross et al., 2015). Timilsina et al. also uncovered only minimal variation among individual gene trees for this arrangement, and so their study effectively resolves this key uncertainty in early monocot phylogeny. Their large collections of nuclear genes also allow them to explore how species-tree inference (e.g., based on concatenated analyses of nuclear genes) may mask substantial conflict among individual gene-trees. For example, a well-supported sister-group arrangement inferred here between the lilioid orders Asparagales and Liliales conflicts with previous plastome-based analyses, which instead place Asparagales as sister to a large clade of commelinid monocots (grasses, palms, gingers and relatives). Timilsina et al. show that incomplete lineage sorting (ILS), which is consistent with rapid diversification among these three major clades, may explain both the nuclear-plastid conflict, and the considerable underlying conflict uncovered among individual nuclear trees concerning this key relationship in monocot phylogeny.

## Understanding morphological trait-based evolution

Monocots display a relatively stable vegetative and reproductive groundplan, typically characterized by linear leaves with parallel venation and trimerous-pentacyclic flowers. Within this framework, trait-based studies help us to understand how diversity has been established across the estimated 60,000-85,000 monocot species. The studies here range from vegetative and branching structure to the structure of the flower, using both traditional and modern techniques. A paper by Choob aims to review and re-evaluate one of the most fundamental features of monocots, the first leaf of the lateral shoot, or prophyll, which is traditionally interpreted as a serial homologue of the cotyledon. Monocots are characterized by a single prophyll, in contrast with a pair of prophylls in other angiosperms. Rejecting an earlier hypothesis that the prophyll evolved by fusion of two phyllomes, Choob notes that the prophyll is highly reduced in some monocots, and outlines an axiomatic “phantom” method for modelling prophyll position and its influence on shoot architecture.

Another aspect of monocot morphology, the inflorescence, was investigated in detail by Martínez-Gómez et al., focusing in particular on the development of the umbellate inflorescence that characterises some members of the orders Asparagales, Alismatales, Dioscoreales and Liliales. They interpreted some umbel-like constructions as formed by a new variation of concaulescence in which axillary buds are displaced relative to the subtending bract by congenital axial fusion (a form of metatopy), in this case in a horizontal position. Their detailed supporting observations are elegantly imaged by both focal stacking of meristems imaged under incident light, and serial sectioning *via* laser ablation tomography and three-dimensional reconstruction. When placed in a phylogenetic context, their results indicate that umbellate inflorescences evolved several times in parallel in monocots, and were derived from a range of different inflorescence types, including cymes and racemes.

Inflorescences composed of spikelets are characteristic of the grass family, Poaceae, which is largely wind-pollinated; they also occur widely in other Poales, including the small neotropical family Rapateaceae, a distinct lineage of Poales with biotically pollinated showy flowers. Koblova et al. employed developmental and anatomical methods to examine the comparative structure of the spikelet and flower in this relatively little-known family. The Rapateaceae spikelet possesses sterile phyllomes on the axis and a single flower that is apparently terminal, in contrast with the spikelet of Poaceae and other Poales, which lack a terminal flower. Thus, the Rapateaceae spikelet could be derived either by extreme reduction from a racemose structure with a modified tip or it could represent a single flower that has undergone a disturbed program of perianth development to produce extra phyllomes below the flower. Gynoecium structure is unusually diverse in Rapateaceae. Koblova et al. hypothesize that the gynoecia of subfamilies Rapateoideae and Monotremoideae, with a single pendent ovule per locule and axial placentation, represent the likely plesiomorphic condition in the family, compared with the derived condition of a fertile plicate carpel zone with numerous ovules, as in subfamily Saxofridericioideae. Postgenital carpel fusion is rare in Poales, occurring only in Bromeliaceae and Rapateaceae. However, this feature has apparently been entirely lost at least twice in Rapateaceae, resulting in the elimination of septal nectaries; most Rapateaceae produce pollen flowers.

Yudina et al. examined comparative floral structure and development in a single monocot genus, *Burmannia* (Burmanniaceae, Dioscoreales), which is unusual in possessing both photosynthetic and mycoheterotrophic species; previous studies are sparse because individual plants are rare and inconspicuous. This study found that its diverse patterns of inflorescence morphology are probably derived from a thyrsoid with two lateral monochasial cymes that represent cincinni. The flowers are elaborate, with a long floral tube consisting of a hypanthium and a perianth tube, with ribs or wings that are morphologically part of the ovary wall at their bases. An interesting and unusual feature highlighted by this study is the synorganization of the stamens and gynoecium into a gynostegium, which in some species involves postgenital fusion between the stamen connectives and the common style, formerly considered to be unique to the eudicot family Apocynaceae.

Finally, the study by Valderrama et al. demonstrates how molecular and morphological approaches can be combined to interrogate the genetic mechanisms underlying adaptive evolution of pollination syndromes in a model system, neotropical *Costus*. The authors used a nuclear targeted-enrichment approach to infer phylogeny, and also performed whole-genome resequencing and transcriptome analysis for 20 closely related species with contrasting pollination syndromes. Their phylogenomic analysis points to multiple, rapid shifts in pollination system across the genus, and indicates previously unknown hybridisation events and possible cryptic species. Valderrama et al. also demonstrate correlated gain and loss of various traits (labellum shape and patterning, and inflorescence and bract colour) in transitions between bird- vs. bee-pollinated systems, but they find no corresponding impact of labellum shape on diversification rates. However, multiple candidate loci related to functional traits involved in pollination display clear signatures of positive selection. Their work points to new avenues for understanding the evolution of pollination syndromes in monocots, and the need for an updated taxonomic revision of the *Costus* group.

## Outlook

Over the past few decades, concerted and integrated studies in monocot character evolution have paralleled increased consensus on monocot phylogenetics. Thus, the large monocot clade represents a promising group for understanding evolutionary patterns and processes. Yet, apart from the highly derived grass family, research in gene function and evolution in flowering plants remains focused primarily on a series of eudicot model species, especially Arabidopsis. The present Research Topic should help to stimulate ongoing research on different monocot species and help unravel the complexities of functional properties in plants.

## Author contributions

All authors listed have made a substantial, direct and intellectual contribution to the work, and approved it for publication.

## Conflict of interest

The authors declare that the research was conducted in the absence of any commercial or financial relationships that could be construed as a potential conflict of interest.

## Publisher’s note

All claims expressed in this article are solely those of the authors and do not necessarily represent those of their affiliated organizations, or those of the publisher, the editors and the reviewers. Any product that may be evaluated in this article, or claim that may be made by its manufacturer, is not guaranteed or endorsed by the publisher.
